# HIV Testing Outcomes Among Blacks or African Americans — 50 Local U.S. Jurisdictions Accounting for the Majority of New HIV Diagnoses and Seven States with Disproportionate Occurrences of HIV in Rural Areas, 2017

**DOI:** 10.15585/mmwr.mm6904a2

**Published:** 2020-01-31

**Authors:** Aba D. Essuon, Hui Zhao, Guoshen Wang, Nicoline Collins, Debra Karch, Shubha Rao

**Affiliations:** ^1^Program Evaluation Branch, Division of HIV/AIDS Prevention, National Center for HIV/AIDS, Viral Hepatitis, STD, and TB Prevention, CDC; ^2^Northrop Grumman, Falls Church, Virginia.

Identifying persons with human immunodeficiency virus (HIV) infection who are unaware of their status and linking them to care are critical steps in achieving viral suppression and reducing the risk for transmitting HIV (*1*). In 2017, 43% of new diagnoses of HIV infection were among persons who self-identify as blacks or African Americans (blacks) (*2*), who represent 13% of the U.S. population (*3*). Fewer blacks, compared with whites, were linked to HIV medical care within 90 days of diagnosis, retained in care, or virally suppressed (*4*). Ending the HIV Epidemic (EHE) is an initiative intended to reduce new HIV infections by 90% from 2020 to 2030 (*5*). EHE’s Phase 1 is focused on 50 jurisdictions* that accounted for >50% of new diagnoses during 2016–2017 and seven states^†^ with disproportionate HIV prevalence in rural areas (*5*). The purpose of this analysis was to examine HIV testing outcomes among blacks in high prevalence EHE jurisdictions, using CDC’s 2017 National HIV Prevention Program Monitoring and Evaluation data. Blacks accounted for 43.2% of CDC-funded tests and 49.1% of new diagnoses of HIV infection. Seventy-nine percent of blacks with newly diagnosed HIV infection were linked to HIV medical care within 90 days (below the 2010 National HIV/AIDS Strategy goal of 85%), 71.4% interviewed for partner services, and 81.8% referred to prevention services. To achieve the goals of EHE, HIV prevention programs should focus on locally tailored evidence-based^§^ testing strategies to enhance and overcome barriers for linkage to and retention in care and reduce onward HIV transmission and HIV-related disparities.

CDC analyzed 2017 HIV testing, linkage to care, and partner services data submitted to the National HIV Prevention Program Monitoring and Evaluation system by 61 CDC-funded health departments^¶^ and 150 CDC-directly funded community-based organizations. Valid HIV tests were those with confirmed HIV-positive test results (discordant and indeterminate test results were excluded). Persons with new diagnoses were those whose HIV test results were positive during the current test and were not previously reported in the health department’s HIV surveillance system or had reported not having had a previous HIV-positive test result. The percentage of positive tests (positivity) for new diagnoses was calculated by dividing the number of new HIV-positive tests by the number of valid tests. Data were stratified by age, gender, race/ethnicity, test setting, U.S. census region, and subpopulation (i.e., men who have sex with men [MSM], persons who inject drugs, heterosexual men, and heterosexual women).** In non–health care settings, data to identify subpopulations were required for all tests conducted; in health care settings data were required only for persons with HIV-positive test results. Subpopulation data included in the analysis were only from non–health care settings.^††^ The following HIV testing outcomes were analyzed among blacks with newly diagnosed HIV infection: linkage to HIV medical care (e.g., attend first medical appointment) within 90 days of positive test result; interview for partner services (i.e., soliciting information from persons with HIV-positive test results regarding sex and drug-injecting partners to notify them of potential exposure and offer services); and referral to HIV prevention services (i.e., behavioral interventions and risk-reduction counseling). Percentages of HIV testing outcomes among blacks were calculated by dividing the number in which “yes” was indicated for the HIV testing outcomes (linked to care within 90 days, interviewed for partner services, and referred to prevention services) by the number of HIV-positive tests. Missing data were excluded from all outcome denominators. SAS (version 9.4; SAS Institute) was used to conduct all analyses.

During 2017, a total of 3,110,049 CDC-funded tests were conducted in the United States, including 1,954,741 (62.9%) in Phase-1 jurisdictions (Table 1). The highest percentages of HIV tests conducted in EHE Phase-1 jurisdictions were among persons aged 20–29 years (36.0%), males (52.3%), and those residing in the South Census region (57.0%). Blacks accounted for 43.2% (844,819) of tests conducted in Phase-1 jurisdictions, twice that of whites (21.6%; 421,656) or Hispanics/Latinos (22.4%; 437,635). Among all new HIV diagnoses, 68.9% (8,154 of 11,843) occurred in Phase-1 jurisdictions, and the highest percentages of new HIV diagnoses were among persons aged 20–29 years (42.9%), males (83.4%), blacks (49.1%), and persons residing in the South Census region (49.1%). The percentage of blacks with newly diagnosed HIV infection (0.5%) was equal to that of Hispanics/Latinos (0.5%) and nearly twice that of whites (0.3%) and Asians (0.3%).

**TABLE 1:** Human immunodeficiency virus (HIV) tests, new diagnoses, and previous diagnoses, by demographic characteristics and subpopulations — CDC-funded Phase-I Ending the HIV Epidemic (EHE) jurisdictions* and other CDC-funded testing sites, United States, 2017

Characteristic	Valid CDC-funded HIV tests		New HIV diagnoses			Previous HIV diagnoses not known to be in care	
	CDC-funded tests	CDC-funded tests, EHE jurisdictions	New diagnoses	New diagnoses, EHE jurisdictions	New HIV positivity, EHE jurisdictions	Previous diagnoses	Previous diagnoses, EHE jurisdictions
	No.	No. (Col %)	No.	No. (Col %)	(%)	No.	No. (Col %)
**Age group (yrs)^†^**							
13–19	218,293	123,447 (6.3)	434	279 (3.4)	0.2	180	142 (1.6)
20–29	1,175,736	704,303 (36.0)	5,221	3,494 (42.9)	0.5	3,345	2,696 (30.2)
30–39	762,944	479,073 (24.5)	3,249	2,322 (28.5)	0.5	3,204	2,467 (27.7)
40–49	416,566	276,244 (14.1)	1,484	1,033 (12.7)	0.4	2,134	1,674 (18.8)
≥50	501,096	355,855 (18.2)	1,411	993 (12.2)	0.3	2,550	1,926 (21.6)
**Gender^§^**							
Male	1,575,493	1,021,993 (52.3)	9,897	6,801 (83.4)	0.7	8,898	6,967 (78.1)
Female	1,498,393	906,192 (46.4)	1,701	1,168 (14.3)	0.1	2,288	1,744 (19.6)
**Test setting**							
Health care facility	2,388,928	1,502,673 (76.9)	7,280	4,863 (59.6)	0.3	8,352	6,754 (75.7)
Non–health care facility	712,278	451,247 (23.1)	4,539	3,290 (40.3)	0.7	3,05	2,159 (24.2)
**U.S. Census region^¶^**							
Northeast	471,609	266,101 (13.6)	1,707	1,216 (14.9)	0.5	1,421	1,015 (11.4)
Midwest	397,121	261,005 (13.4)	1,661	1,136 (13.9)	0.4	1,765	1,589 (17.8)
South	1,792,105	1,114,464 (57.0)	6,108	4,007 (49.1)	0.4	7,283	5,530 (62.0)
West	416,921	303,177 (15.5)	2,144	1,691 (20.7)	0.6	827	722 (8.1)
U.S. dependent areas	32,293	9,994 (0.5)	223	104 (1.3)	1.0	131	61 (0.7)
Race/Ethnicity**							
White	819,524	421,656 (21.6)	2,330	1,378 (16.9)	0.3	2,104	1,516 (17.0)
Black or African American	1,257,198	844,819 (43.2)	5,622	4,007 (49.1)	0.5	6,403	5,214 (58.5)
Hispanic or Latino	677,954	437,635 (22.4)	2,974	2,076 (25.5)	0.5	1,913	1,359 (15.2)
Asian	73,379	53,066 (2.7)	223	164 (2.0)	0.3	133	112 (1.3)
AI/AN	16,269	7,519 (0.4)	59	27 (0.3)	0.4	39	24 (0.3)
H/PI	6,509	4,176 (0.2)	15	13 (0.2)	0.3	14	12 (0.1)
Multirace	23,935	12,497 (0.6)	134	90 (1.1)	0.7	76	51 (0.6)
**Subpopulation in non–health care setting^††^**							
MSM	180,748	123,635 (27.4)	3,175	2,326 (70.7)	1.9	1,602	1,204 (55.8)
Transgender persons	7,763	5,377 (1.2)	109	89 (2.7)	1.7	70	55 (2.5)
Persons who inject drugs	38,190	17,142 (3.8)	145	98 (3.0)	0.6	121	68 (3.1)
Heterosexual men	173,259	100,521 (22.3)	443	305 (9.3)	0.3	424	265 (12.3)
Heterosexual women	182,852	108,053 (23.9)	376	262 (8.0)	0.2	350	248 (11.5)
**Total**	**3,110,049**	**1,954,741 (100.0)**	**11,843**	**8,154 (100.0)**	**0.4**	**11,427**	**8,917 (100.0)**

**Abbreviations:** AI/AN = American Indian or Alaska Native; Col = column; H/PI = Native Hawaiian or Pacific Islander; MSM = gay, bisexual, and other men who have sex with men.

* Fifty local jurisdictions accounting for >50% of new diagnoses during 2016–2017 and seven states with disproportionate occurrence of HIV in rural areas.

^†^ For age, the numbers of records missing or invalid are as follows: in the columns “Valid CDC-funded HIV tests,” 35,414 (1.1%) of all CDC-funded valid HIV tests and 15,819 (0.8%) of valid HIV tests in EHE jurisdictions; in the columns under “New HIV diagnoses,” 44 (0.4%) of total new diagnoses and 33 (0.4%) of new diagnoses in EHE jurisdictions; in the columns under “Previous HIV diagnoses,” 14 (0.1%) of total previous diagnoses and 12 (0.1%) of previous diagnoses in EHE jurisdictions.

^§^ For gender, the numbers of records reported as transgender, missing, or invalid are as follows: in the columns under “Valid CDC-funded HIV tests,” 36,163 (1.2%) of all CDC-funded valid HIV tests and 26,556 (1.4%) of valid HIV tests in EHE jurisdictions; in the columns under “New HIV diagnoses,” 245 (2.1%) of total new diagnoses and 185 (2.3%) of new diagnoses in EHE jurisdictions; in the columns under “Previous HIV diagnoses,” 241 (2.1%) of total previous diagnoses and 206 (2.3%) of previous diagnoses in EHE jurisdictions.

^¶^ *Northeast*: Connecticut, Maine, Massachusetts, New Hampshire, New Jersey, New York, Pennsylvania, Rhode Island, and Vermont; *Midwest*: Illinois, Indiana, Iowa, Kansas, Michigan, Minnesota, Missouri, Nebraska, North Dakota, Ohio, South Dakota, and Wisconsin; *South*: Alabama, Arkansas, Delaware, District of Columbia, Florida, Georgia, Kentucky, Louisiana, Maryland, Mississippi, North Carolina, Oklahoma, South Carolina, Tennessee, Texas, Virginia, and West Virginia; *West*: Alaska, Arizona, California, Colorado, Hawaii, Idaho, Montana, Nevada, New Mexico, Oregon, Utah, Washington, and Wyoming.

** For race/ethnicity, the numbers of records missing or invalid are as follows: in the columns under “Valid CDC-funded HIV tests,” 235,281 (7.6%) of all CDC-funded valid HIV tests and 173,373 (8.9%) of valid HIV tests in EHE jurisdictions; in the columns under “New HIV diagnoses,” 486 (4.1%) of total new diagnoses and 399 (4.9%) of new diagnoses in EHE jurisdictions; in the columns under “Previous HIV diagnoses,” 745 (6.5%) of total previous diagnoses and 629 (7.1%) of previous diagnoses in EHE jurisdictions.

^††^ MSM include males who reported male-to-male sexual contact as well as males who reported both male-to-male sexual contact and injection drug use in the past 12 months. Persons who inject drugs include persons who reported injection drug use in the past 12 months. Heterosexual males include males who only reported sexual contact with a female in the past 12 months. Heterosexual females include females who only reported sexual contact with a male in the past 12 months. Data on subpopulations were limited to those tested in non–health care settings. For subpopulation in non–health care settings, the numbers of records missing or invalid are as follows: in the columns under “Valid CDC-funded HIV tests,” 129,466 (18.2%) of all CDC-funded valid HIV tests and 96,519 (21.4%) of valid HIV tests in EHE jurisdictions; in the columns under “New HIV diagnoses,” 291 (6.4%) of total new diagnoses and 210 (6.4%) of new diagnoses in EHE jurisdictions; in the columns under “Previous HIV diagnoses,” 483 (15.8%) of total previous diagnoses and 319 (14.8%) of previous diagnoses in EHE jurisdictions. Totals for subpopulation in non–health care settings are 712,278 for all CDC-funded HIV tests, 451,247 for CDC-funded tests in EHE jurisdictions, 4,539 for total new HIV diagnoses, 3,290 for new diagnoses in EHE jurisdictions, 3,050 for previously diagnosed, and 2,159 for previously diagnosed in EHE jurisdictions.

In 2017 CDC-funded testing programs identified 11,427 persons with a previous diagnosis of HIV infection who were not known to be in care, 8,917 (78.0%) of whom were in Phase-1 jurisdictions. Persons with a previous diagnosis who were not known to be in care were predominately aged 20–29 years (30.2%) or 30–39 years (27.7%), male (78.1%), black (58.5%), and residents of the South Census region (62.0%). The number of blacks in Phase-1 jurisdictions with a previous diagnosis and not known to be in care (5,214; 58.5%) was more than three times that of whites (1,516; 17.0%) and Hispanics/Latinos (1,359; 15.2%).

Among the 844,819 blacks tested in Phase-1 jurisdictions in 2017, 37.7% (318,835) were persons aged 20–29 years; 49.7% were males; and 63.2% were persons residing in the South (Table 2). Of the 4,007 blacks who received a new diagnosis of HIV infection, the percentage positivity was highest among persons aged 20–29 years (0.6%), males (0.7%), and persons residing in the West Census region (0.7%). Among blacks who received a new diagnosis, 79.2% were linked to care within 90 days, 71.4% were interviewed for partner services, and 81.8% were referred to HIV prevention services. By region, linkage of blacks with newly diagnosed HIV infection to medical care within 90 days was lowest in the West (71.7%), whereas the lowest percentages of partner services interviews (58.9%) and referrals to HIV prevention services (70.2%) were in the Midwest. By subpopulation, the highest percentage of tests conducted in EHE jurisdictions were among MSM (27.4%) (Table 1), who also had the highest rates of HIV-positive test results (3.3%) among subpopulation blacks (Table 2).

**TABLE 2:** Linkage to human immunodeficiency virus (HIV) medical care for blacks or African Americans (blacks) with newly diagnosed HIV infection — CDC-funded Phase-I Ending the HIV Epidemic (EHE) jurisdictions,* United States, 2017

Characteristic	No. (%)				
	CDC-funded tests among blacks in EHE jurisdictions	New diagnoses among blacks in EHE jurisdictions	Linked to HIV medical care within 90 days of diagnoses	Interviewed for partner services	Referred to HIV prevention services
**Age group at test (yrs)^†^**					
13–19	59,683	183 (0.3)	117 (84.2)	88 (71.0)	122 (84.7)
20–29	318,835	1,869 (0.6)	1,257 (81.3)	1,041 (74.6)	1,301 (83.2)
30–39	193,353	1,013 (0.5)	685 (78.8)	549 (70.7)	679 (81.1)
40–49	110,772	429 (0.4)	262 (74.4)	215 (70.0)	267 (78.1)
≥50	158,462	501 (0.3)	301 (74.5)	225 (62.0)	322 (80.7)
**Gender^§^**					
Male	419,746	3,124 (0.7)	2,039 (79.2)	1,660 (71.8)	2,124 (82.4)
Female	420,203	785 (0.2)	517 (78.6)	407 (69.8)	499 (79.6)
**Test setting**					
Health care facility	634,620	2,420 (0.4)	1,644 (80.2)	1,370 (72.7)	1,554 (81.7)
Non–health care facility	209,843	1,587 (0.8)	980 (77.6)	749 (69.2)	1,139 (82.0)
**U.S. Census region^¶^**					
Northeast	115,415	586 (0.5)	447 (86.3)	384 (83.8)	473 (83.9)
Midwest	139,838	675 (0.5)	430 (77.0)	331 (58.9)	448 (70.2)
South	534,304	2,378 (0.4)	1,539 (79.0)	1,200 (74.3)	1,570 (86.6)
West	55,260	368 (0.7)	208 (71.7)	204 (61.1)	202 (73.2)
U.S. dependent areas	2	0 (0.0)	0 (0.0)	0 (0.0)	0 (0.0)
Subpopulation in non–health care setting**					
MSM	31,508	1,030 (3.3)	664 (80.6)	484 (71.3)	763 (84.2)
Transgender	1,964	52 (2.6)	34 (79.1)	28 (73.7)	41 (85.4)
Persons who inject drugs	3,139	26 (0.8)	8 (42.1)	9 (47.4)	18 (72.0)
Heterosexual males	56,676	186 (0.3)	113 (74.8)	97 (65.1)	132 (80.0)
Heterosexual females	64,160	189 (0.3)	114 (75.0)	92 (74.8)	125 (77.6)
**Total**	**844,819**	**4,007 (0.5)**	**2,624 (79.2)**	**2,119 (71.4)**	**2,693 (81.8)**

**Abbreviation:** MSM = gay, bisexual, and other men who have sex with men.

* Fifty local jurisdictions accounting for >50% of new diagnoses during 2016–2017 and seven states with disproportionate occurrence of HIV in rural areas.

^†^ For age, the numbers of records missing or invalid are as follows: in the column “CDC-funded tests among blacks in EHE jurisdictions,” 3,714 (0.4%); in the column “New HIV diagnoses among blacks in EHE jurisdictions,” 12 (0.3%); in the column “Linked to HIV medical care within 90 days of diagnoses,” 2 (0.1%); in the column “Interviewed for partner services,” 1 (0.05%); in the column “Referred to HIV prevention services,” 2 (0.1%).

^§^ For gender, the numbers of records reported as transgender, missing, or invalid are as follows: in the column “CDC-funded tests among blacks in EHE jurisdictions,” 4870 (0.6%); in the column “New HIV diagnoses among blacks in EHE jurisdictions,” 98 (2.4%); in the column “Linked to HIV medical care within 90 days of diagnoses,” 68 (2.6%); in the column “Interviewed for partner services,” 52 (2.5%); in the column “Referred to HIV prevention services,” 70 (2.6%).

^¶^ *Northeast*: Connecticut, Maine, Massachusetts, New Hampshire, New Jersey, New York, Pennsylvania, Rhode Island, and Vermont; *Midwest*: Illinois, Indiana, Iowa, Kansas, Michigan, Minnesota, Missouri, Nebraska, North Dakota, Ohio, South Dakota, and Wisconsin; *South*: Alabama, Arkansas, Delaware, District of Columbia, Florida, Georgia, Kentucky, Louisiana, Maryland, Mississippi, North Carolina, Oklahoma, South Carolina, Tennessee, Texas, Virginia, and West Virginia; *West*: Alaska, Arizona, California, Colorado, Hawaii, Idaho, Montana, Nevada, New Mexico, Oregon, Utah, Washington, and Wyoming.

** MSM include males who reported male-to-male sexual contact as well as males who reported both male-to-male sexual contact and injection drug use in the past 12 months. Persons who inject drugs include persons who reported injection drug use in the past 12 months. Heterosexual males include males who only reported heterosexual contact with a female in the past 12 months. Heterosexual females include females who only reported heterosexual contact with a male in the past 12 months. Data on behavioral risk factors used to define the subpopulation were limited to those tested in non–health care settings. For subpopulation in non–health care settings, the numbers of records missing or invalid are as follows: in the column “CDC-funded tests among blacks in EHE jurisdictions,” 52,396 (25.0%); in the column “New HIV diagnoses among blacks in EHE jurisdictions,” 104 (6.6%); in the column “Linked to HIV medical care within 90 Days of diagnoses,” 47 (4.8%); in the column “Interviewed for partner services,” 39 (5.2%); in the column “Referred to HIV prevention services,” 60 (5.3%). Totals for subpopulation in non–health care settings are 209,843 for CDC-funded tests among blacks in EHE jurisdictions, 1,587 for new diagnoses among blacks in EHE jurisdictions, 980 for linked to HIV medical care within 90 days of diagnoses, 749 for interviewed for partner services, and 1,139 for referred to HIV prevention services.

Black MSM accounted for 15.0% (31,508 of 209,843) of tests and 64.9% (1,030 of 1,587) of new HIV diagnoses in non–health care settings. More than 70% of black MSM with newly diagnosed HIV infection were linked to HIV medical care (80.6%), interviewed for partner services (71.3%), or referred to HIV prevention services (84.2%) (Table 2).

## Discussion

The goal of HIV testing programs is to identify persons with HIV infection who are unaware of their status and to link all persons with HIV-positive test results to services. In 2017, 62.9% of CDC-funded tests and 68.9% of new diagnoses of HIV infection were in Phase-1 jurisdictions, among whom blacks accounted for >40% of tests (40.4%) and new diagnoses (47.5%). Blacks also accounted for 58.5% of persons with a previous diagnosis not known to be in care. Compared with whites, a higher percentage of blacks in Phase-1 jurisdictions received a new diagnosis (49.1%) or had previously received a diagnosis and were not known to be in care (58.5%).

This analysis found that HIV testing services supported by CDC funding are an important resource for identifying persons with new and previously diagnosed HIV infection who are not in care, especially in Phase-1 jurisdictions. Testing sites in Phase-1 jurisdictions are especially critical for blacks, who account for the largest numbers of persons tested, new diagnoses of HIV infection, and persons previously diagnosed not known to be in care. Factors such as stigma, comorbidities, and socioeconomic inequalities might increase blacks’ risk for acquiring or transmitting HIV and limit access to quality health care, housing, and HIV prevention messaging (*3*). Delayed entry into HIV prevention and treatment, especially among blacks, leads to worse HIV care outcomes (e.g., delayed linkage to care and viral suppression) (*6*). Although 79.2% of blacks with newly diagnosed HIV infection in Phase-1 jurisdictions were linked to HIV medical care within 90 days, this percentage was below the 2010 National HIV/AIDS Strategy (NHAS) goal of 85% (*7*). This outcome suggests that the 2020 NHAS goals of 85% linkage within 30 days of diagnosis (*7*) and the EHE initiative to reduce new HIV infections by 90% by 2030 (*7*) might be challenging to achieve among blacks without expanding current efforts and implementing novel testing strategies.

The findings in this report are subject to at least four limitations. First, the findings are based on data from CDC-funded tests, which are not representative of all U.S. HIV testing. Second, estimates of persons with newly diagnosed HIV infection rely on verification using CDC’s HIV surveillance data or self-report, which could result in an overestimation of new diagnoses. Third, data on linkage to HIV medical care, interview for partner services, and referral to HIV prevention services exclude missing data from the denominator and likely overestimate the percentage of persons receiving services. Finally, data on subpopulations are collected for all tests in non–health care settings but only for HIV-positive tests in health care settings, resulting in underreporting of tests among subpopulations. 

CDC-funded HIV testing programs are identifying new and previously diagnosed HIV infections in persons not known to be in care in Phase-1 jurisdictions, but challenges linking persons with new and previously diagnosed infections to care differ (*8*). Broader implementation of routine HIV screening and HIV-related services, most notably among black MSM, has critical public health implications. To achieve the goals of the EHE initiative, HIV prevention programs will need to focus on locally tailored evidence-based testing strategies to overcome barriers for and enhance linkage to and retention in care, provide prophylaxis and treatment, and reduce onward HIV transmission and HIV-related disparities.

**Summary**

**What is already known about this topic?**

Ending the HIV Epidemic (EHE) jurisdictions are disproportionately affected by human immunodeficiency virus (HIV).

**What is added by this report?**

In 2017, blacks accounted for >40% of those tested and new diagnoses in EHE jurisdictions. Compared with whites, more blacks in EHE jurisdictions received a new diagnosis or were identified as a person with previously diagnosed HIV infection.

**What are the implications for public health practice?**

HIV prevention programs focused on locally tailored innovative testing, linkage, reengagement, and prophylaxis and treatment for blacks could help to achieve the national goals to end the HIV epidemic in the United States.

## Footnotes

*
https://www.hiv.gov/federal-response/ending-the-hiv-epidemic/overview .

† Seven states: Alabama, Arkansas, Kentucky, Mississippi, Missouri, Oklahoma, and South Carolina.

§ https://effectiveinterventions.cdc.gov; https://www.cdc.gov/hiv/research/interventionresearch/compendium/lrc/index.html.

¶ CDC-funded health departments include 50 states, the District of Columbia, Puerto Rico, the U.S. Virgin Islands, and the following eight metropolitan statistical areas or specified metropolitan divisions: Baltimore, Chicago, Fulton County (Atlanta), Houston, Los Angeles County, New York City, Philadelphia, and San Francisco.

** MSM includes males who reported male-to-male sexual contact; and males who reported both male-to-male sexual contact and injection drug use in the past 12 months. Persons who inject drugs include persons who reported injection drug use in the past 12 months. Heterosexual men includes males who only reported heterosexual contact with a female in the past 12 months. Heterosexual women includes females who only reported heterosexual contact with a male in the past 12 months.

†† Non–health care settings include HIV testing sites; community setting (various, shelter/transitional housing, syringe services program, other); correctional facilities (non–health care); health department (field visit); and other non–health care.
